# Phenotypic Mapping of The Chicken Embryonic Thymic Microenvironment Developing Within an Organ Culture System

**DOI:** 10.1155/1993/51718

**Published:** 1993

**Authors:** Natalie J. Davidson, Richard L. Boyd

**Affiliations:** Department of Pathology and Immunology Monash University Medical School Commercial Road Prahran 3181 Australia

**Keywords:** Chicken embryonic thymus, thymic stromal cells, thymus organ culture

## Abstract

The chicken thymic microenvironment, as it developed in an embryonic thymus organ culture system, was phenotypically mapped using a panel of mAb defining both epithelial and nonepithelial stromal cell antigens. We have previously reported that thymocyte proliferation and differentiation will proceed for up to 6–8 days in thymus organ culture, hence demonstrating the functional integrity of the thymic microenvironment *in vitro*. During this time, the stromal component reflected that of the normal embryo with cortical and medullary epithelial areas readily identifiable by both morphology and surface-antigen expression. An abundance of subcapsular and cortical epithelial antigens was detected in the cultured thymus, particularly those normally expressed by the epithelium lining the capsule, trabeculae, and vascular regions (type epithelium) in the adult and embryonic thymus. Medullary epithelial antigens developed in organ culture, although were present in lower frequency than observed in the age-matched embryonic thymus. MHC class II expression by both epithelial and nonepithelial cells was maintained at high levels throughout the culture period. With increasing time in culture, the ratio of epithelial to nonepithelial cells decreased, concurrent with a decrease in thymocyte frequency and suggestive of a bidirectional interaction between these two cell types. Thus, a functionally intact thymic microenvironment appears to be maintained in embryonic thymus organ culture, a model that is currently being exploited to assess the role of stromal antigens, as defined by our mAb, in the process of thymopoiesis.


*Developmental Immunology*, 1993, Vol. 3, pp. 147–158
Reprints available directly from the publisher
Photocopying permitted by license only

© 1993 Harwood Academic Publishers GmbH
Printed in the United States of America

### Phenotypic Mapping of the Chicken Embryonic Thymic Microenvironment Developing within an Organ Culture System

NATALIE J. DAVIDSON* and RICHARD L. BOYD

*Department of Pathology and Immunology, Monash University Medical School, Commercial Road, Prahran, 3181, Australia*

The chicken thymic microenvironment, as it developed in an embryonic thymus organ culture system, was phenotypically mapped using a panel of mAb defining both epithelial and nonepithelial stromal cell antigens. We have previously reported that thymocyte proliferation and differentiation will proceed for up to 6–8 days in thymus organ culture, hence demonstrating the functional integrity of the thymic microenvironment *in vitro*. During this time, the stromal component reflected that of the normal embryo with cortical and medullary epithelial areas readily identifiable by both morphology and surface-antigen expression. An abundance of subcapsular and cortical epithelial antigens was detected in the cultured thymus, particularly those normally expressed by the epithelium lining the capsule, trabeculae, and vascular regions (type I epithelium) in the adult and embryonic thymus. Medullary epithelial antigens developed in organ culture, although were present in lower frequency than observed in the age-matched embryonic thymus. MHC class II expression by both epithelial and nonepithelial cells was maintained at high levels throughout the culture period. With increasing time in culture, the ratio of epithelial to nonepithelial cells decreased, concurrent with a decrease in thymocyte frequency and suggestive of a bidirectional interaction between these two cell types. Thus, a functionally intact thymic microenvironment appears to be maintained in embryonic thymus organ culture, a model that is currently being exploited to assess the role of stromal antigens, as defined by our mAb, in the process of thymopoiesis.

KEYWORDS: Chicken embryonic thymus, thymic stromal cells, thymus organ culture.

### INTRODUCTION

The thymus is comprised of a heterogeneous array of stromal cells and cytokines, both stromal- and thymocyte-derived, that constitute a microenvironment essential for the production of a competent T-cell repertoire (reviewed in Boyd and Hugo, 1991; Carding et al., 1991). An essential step in T-cell differentiation involves the interaction between stromal cell MHC antigens and T-cell receptor (TcR) on developing thymocytes (Zijlstra et al., 1990; Cosgrove et al., 1991). However, lymphostromal interactions involving non-MHC molecules, such as those occurring prior to T-cell TcR expression on immature thymocytes, while equally important, are less well-defined. Clearly, there is a need to further define and characterize thymic stromal-cell subsets/surface antigens and their specific role in the various stages of T-cell differentiation.

Monoclonal antibodies (mAb) raised against thymic stromal elements of the mouse, rat, and human have revealed a complex phenotypic profile, indicating the antigenically distinct regions within and between the basic cortical-medullary definition of the thymic stroma (Haynes, 1984; van Vliet et al., 1985; Godfrey et al., 1990; Izon and Boyd, 1990; reviewed in Kampinga et al., 1989; Brekelmans and van Ewijk, 1990). Similarly,

*Corresponding author.

mAb reactive with chicken stromal elements (Boyd et al., 1992), including MHC class II-specific reagents (Guillemot et al., 1984; Boyd et al., 1992), have delineated a similar stromal heterogeneity, demonstrating the conserved structural and antigenic nature of the thymus between the avian and mammalian species. Amongst the mAb produced in our laboratory (Boyd et al., 1992) are those specific for epithelium lining the capsule, trabeculae, and perivascular regions (type I epithelium, as defined by electron microscopy [van de Wijngaert et al., 1984]). Such reagents are distinct from those that label both the subcapsular and medullary epithelium described in various mammalian species (Haynes et al., 1984; Colic et al., 1988; Godfrey et al., 1990; Izon and Boyd, 1990), in addition to the chicken (Boyd et al., 1992). Takeuchi et al. (1991) have recently described a mAb reacting only with subcapsular epithelium in the human thymus; however, on close examination, it appears that this mAb also shows significant, although weak, reactivity with cortical epithelium. The type I epithelium-specific determinants facilitate further analysis of this region of the thymus and are of particular interest as they are selectively deficient in L200 chickens that develop autoimmune scleroderma (Boyd et al., 1991).

Thymic stromal cell-reactive mAb provide a means with which to not only map the thymic microenvironment, but to examine the functional contribution of mAb-defined thymic stromal subsets to thymocyte differentiation. One method by which this may be achieved is through addition of mAb to embryonic thymus organ culture (ETOC) and monitoring the subsequent effects upon thymocyte development. In a previous report (Davidson et al., 1992), we detailed the growth kinetics and phenotypic development of thymocytes in chicken ETOC. In this model, as for murine fetal thymus organ culture (FOTC) (reviewed in Jenkinson and Owen, 1990), thymocyte proliferation and differentiation of both $\alpha\beta$ and $\gamma\delta$ TcR lineage cells proceed in a manner paralleling that in the embryo. It follows, therefore, that the essential components of the thymic microenvironment must develop and be functionally maintained in ETOC. In one of very few studies, van Vliet et al. (1985) have shown, through the use of specific mAb, that both cortical and medullary epithelial cells are represented in cultured fetal mouse thymus. Similarly, rat thymus fragments cultured *in vitro* are composed of cells reactive with mAb that normally stain either cortical or medullary epithelium (Kendall et al., 1988). Hence, as a prelude to the functional assessment of thymic stromal subsets in ETOC, their *in vitro* development was mapped using a panel of mAb (Boyd et al., 1992) and compared to that *in ovo*. The phenotypic development of stromal subsets in the normal chicken embryo have recently been described (Wilson et al., 1992).

### RESULTS

The results are categorized according to cell-type antigens recognized by the mAb: epithelial cell antigens; antigens shared between epithelial and nonepithelial cells; and nonepithelial cell antigens (Tables 1–3 and Figs. 1–3, respectively). Thymic epithelium-reactive mAb have, in some instances, been categorized according to CTES (Clusters of Thymic Epithelium Staining) guidelines as defined in Kampinga et al. (1989).

### Epithelial Cell Antigens (Table 1)

All epithelial tissue (as defined by double-labeling with an anticytokeratin reagent) expressed an antigen defined by MUI-54 (CTES XX.a) during thymic development *in ovo*. Both of these panepithelial reagents defined cortical- and medullarylike epithelium in the cultured thymus, particularly from days 2–8. Subsets of flattened type I epithelium lining the subcapsule, subtrabeculae, and perivascular regions were defined by MUI-53 in the embryonic thymus, but showed an unusual distribution in ETOC. At days 4–6 ETOC, as much as half of the epithelium was stained with this mAb, including some indentifiable subcapsular epithelium (Fig. 1a). By days 10–12, the majority of epithelium expressed this marker. Such reactivity revealed a lack of type I epithelial organization in the cultured thymus when compared to that observed in the normal embryo (Fig. 1c). Isolated cortical epithelial cells of the embryo/adult thymus were defined by MUI-52 (Fig. 1g). A marked increase in the frequency of epithelial expression of this molecule was observed in ETOC. Isolated epithelial cells stained with MUI-52 at the onset of culture (10E), approximately 50% by days 4–6 ETOC (Fig. 1e)

TABLE 1
Development of Epithelial Cell-Specific Antigens in ETOC in Comparison to Age-Matched Embryos[a]

| mAb[b] | Adult thymus localization | Day 0 (10E) ETOC | Days 4–6[c] ETOC | Days 10–12[c] ETOC | 14–20 E[d] |
|---|---|---|---|---|---|
| MUI-54 (CTES XX.a) | Pan Ep | Pan Ep | PanEp | Pan Ep | Adult localization |
| MUI-51, 53, 70 | SC/STb and M PV Ep | Isolated Ep (51, 70) and 30–50% Ep (53) | Isolated Ep (51, 70) and 30–50% Ep (53) | Majority Ep and isolated KNC | Subset Ep |
| MUI-52 | C Ep | Isolated Ep | ~50% Ep | Majority Ep and subset KNC | Isolated Ep |
| MUI-62 (CTES II) | M Ep | Rare, isolated Ep | Isolated Ep clusters | Isolated Ep clusters | Isolated Ep clusters |
| MUI-58 (CTES V.c) | SC and M Ep | Isolated Ep | Up to 50% Ep | Majority Ep | Subset Ep |

[a]Thymus sections were screened with each mAb at 2-day intervals from days 0–12 ETOC and from 10E to 1 day posthatch.
[b]mAb are classified according to CTES nomenclature where the appropriate category has been defined (Kampinga et al., 1989).
[c]Results are similar between these time points and hence grouped for simplicity.
[d]No change in mAb distribution >14E but for increased extent with developmental age.
Abbreviations: C: cortex; Ep: epithelium; ETOC: embryonic thymus organ culture; KNC: keratin-negative cells; M: medulla; PV: perivascular; SC: subcapsular; STb: subtrabecular.

and the majority by days 10–12. Isolated, or small clusters of, medullary epithelial cells are defined by the mAb MUI-62 (CTES II) in the normal adult thymus, the pattern first observed beginning at 10–12E. These cells were present throughout the ETOC period, although at a reduced frequency compared to the *in ovo* thymus. A phenotypic relationship between subcapsular and medullary epithelium is demonstrated by the mAb MUI-58 (CTES V.c) in the adult thymus. A subset of these cells express this marker during embryogenesis, increasing to the adult distribution by hatching (Fig. 1k). In ETOC, MUI-58 reacted with epithelial cells, including subcapsular epithelium at days 2–4 (Fig. 1i), the frequency of reactive cells increasing with time in culture to encompass most epithelium by days 10–12.

The combined reactivity of these mAb and the anticytokeratin reagent, facilitated the mapping of epithelial cell development in ETOC. Cortical- and medullarylike epithelial areas, recognized by their morphology and antigenic profile, were clearly identifiable until day 6 ETOC (e.g., Fig. 1b). Thereafter, epithelial cell regions appeared to condense such that by days 10–12 dense epithelial areas were surrounded by keratin-negative tissue, the latter comprising at least half of the lobe (data not shown). Additionally, it is interesting to note the reactivity of MUI-53 and MUI-52 with a subset of nonepithelial (keratin-negative) cells at this stage, staining that has not been previously noted *in vivo*.

### Antigens Shared Between Epithelial and Nonepithelial Cells (Table 2)

Scattered epithelial and nonepithelial cells (reticular fibroblasts and macrophages) in the cortex, medulla, and within the trabeculae and perivascular spaces are defined by MUI-66, although reactivity is predominantly directed toward clusters of medullary epithelial cells (Fig. 2c). Such distribution was detectable from 14E through to the adult. At the onset of culture (10E), all epithelium expressed this marker and remained positive throughout the culture period, including both cortical- and medullarylike epithelium (Fig. 2a). The majority of keratin-negative cells also strongly expressed this molecule in ETOC, present as isolated cells or small clusters within epithelial areas and as large areas around the epithelium. This nonepithelial reactivity expanded as the proportion of keratin-negative tissue increased with time in culture. In the embryo, isolated medullary epithelial clusters and keratin-negative cells scattered throughout the thymus were stained with MUI-72 and MUI-80 (Fig. 2k), although, the keratin-negative reactivity of MUI-72 was restricted to the medulla, and MUI-80 stained isolated epithelial cells, showing extensive granular reactivity. In cultured thymus lobes, isolated, medullarylike epithelium and keratin-negative cells were recognized by these markers; however, the determinant defined by MUI-72 was not detectable until days 4–6 of culture. MUI-80 also showed a granular reactivity over much of the lobe (Fig. 2i). A monomorphic MHC class II determinant is recognized by MUI-78 (Boyd et al., 1992), associated with isolated epithelial cells in the thymic cortex and the majority of epithelial and nonepithelial cells in the medulla of the adult (Fig. 2g). This pattern of expression was noted from 14–16E, and prior to this was associated with isolated epithelial and nonepithelial cells, as cortical-medullary delineation is not morphologically defined until 14E. Up to day 6 ETOC, this distribution of MHC class II expression developed and was maintained much like that in the embryo (Fig. 2e). By days 10–12, however, the majority of epithelial and nonepithelial cells were MHC class II positive, and, in most cases, MUI-78 showed confluent reactivity over the entire lobe with a granular appearance characteristic of a secreted molecule (data not shown).

### Nonepithelial Cell Antigens (Table 3)

Connective tissue fibroblasts associated with the capsule, trabecular, perivascular spaces, within keratin-negative areas, and isolated cells in the medulla are recognized by MUI-56. At the onset of culture, there was a perithymic distribution of this connective tissue. Infrequent nonepithelial cells within epithelial areas also expressed this marker. At days 4–6 ETOC, MUI-56$^{+}$ keratin-negative cells were present both within and around epithelial areas (Fig. 3a), similar to the embryo (Fig. 3b), and expanded to form at least half of the tissue by days 10–12. Macrophages (MΦ) primarily located in the adult thymus medulla, keratin-negative areas, and trabeculae are recognized by MUI-79. These cells were undetectable at the onset of ETOC, but present at low frequency by days 4–6, increasing by days 10–12. Medullary, but not cortical, vascular endothelium defined by MUI-82 in the adult and observed from 14–16E was absent throughout the ETOC period.

Thymocyte development was examined in cultured thymus sections in relation to the development of the stroma. MUI-36 reacted with B cells and a subset of thymocytes and MΦ located in the medulla of the adult thymus (Fig. 3f) (Boyd et al., 1992; A. G. Bean, N. J. Davidson, H. A. Ward and R. L. Boyd, in preparation). In the 10E thymus, isolated cells expressed this marker, located within and around the lobe. During the organ culture period, the majority of thymocytes expressed this marker (Fig. 3d), a significant increase over that in the embryo (peaking at 61% by day 8 ETOC in contrast to 28% at 16E; data not shown). A subset of MΦ was stained with MUI-36 in ETOC; however, no B cells were detectable in this system as staining with goat anti-chicken Ig (reactive with heavy and light chain) was

TABLE 2
Development of Antigens Shared between Epithelial and Nonepithelial Cells in ETOC in Comparison to Age-Matched Embryos[a]

| mAb | Adult thymus localization | Day 0 (10E) ETOC | Days 4–6[b] ETOC | Days 10–12[b] ETOC | 14–20E[c] |
|---|---|---|---|---|---|
| MUI-66 | M Ep clusters, isolated K+/− in C, M, KNA, Tb, PVS | Pan Ep | Pan Ep and majority KNC (intense) | Pan Ep and majority KNC (intense) | Adult localization |
| MUI-72 | M Ep clusters, isolated KNC in M and KNA | Negative | Isolated Ep and KNC | Isolated Ep and KNC | Isolated Ep and non-Ep |
| MUI-78 (MHC class II) | isolated C Ep, majority M Ep, and non-Ep (KNA) | Isolated Ep and KNC | Isolated C-like Ep, confluent M-like Ep, and associated KNC | Majority Ep and KNC (confluent in some lobes) | Adult localization |
| MUI-80 | M Ep clusters, isolated K+/− in C, M, KNA, Tb, PVS | Isolated Ep (midlobe) | Isolated M-like Ep; granular reactivity over most of lobe | Isolated Ep and majority KNC (intense, granular) | Isolated Ep, KNC[d] in KNA and Tb (granular) |

[a]Thymus sections were screened with each mAb at 2-day intervals from days 0–12 ETOC and from 10E to 1 day posthatch.
[b]Results are similar between these time points and are hence grouped for simplicity.
[c]No change in mAb distribution >14E but for somewhat increased extent with increasing developmental age.
[d]KNC reactivity present from 16E.
Abbreviations: C: cortex; Ep: epithelium; ETOC: embryonic thymus organ culture; KNC/KNA: keratin-negative cells/areas; M: medulla; PVS: perivascular space; Tb: trabeculae.

TABLE 3
Development of Nonepithelial Cell Antigens in ETOC in Comparison to Age-Matched Embryos[a]

| mAb | Adult thymus localization | Day 0 (10E) ETOC | Days 4–6[b] ETOC | Days 10–12[b] ETOC | 14–20E[c] |
|---|---|---|---|---|---|
| MUI-56 | CT associated with Cap, PVS, KNA, and within M Ep areas | Perithymic CT and isolated KNC within Ep areas | CT/KNC within and around Ep areas (intense) | Large areas KNC within and around Ep areas (intense) | Adult localization |
| MUI-79 | Isolated MΦ in M, KNA, and Tb | Negative | Rare, isolated KNC | Isolated KNC | Adult localization |
| MUI-82 | Vascular endothelium (mainly M) | Negative | Negative | Negative | Adult localization |
| MUI-36 | C: Th (weak); M: subset Th and MΦ B cells | Rare, isolated Th within and around Ep lobes | Majority Th in C-like Ep areas and isolated KNC | Isolated Th in Ep and KNA | Adult localization |
| MUI-83 | C (intense) >M Th and isolated Th in KNA | Isolated Th | Majority Th (intense) | Isolated Th in Ep and KNA | Adult localization |

[a]Thymus sections were screened with each mAb at 2-day intervals from days 0–12 ETOC and from 10E to 1 day posthatch.
[b]Results are similar between these time points and are hence grouped for simplicity.
[c]No change in mAb distribution >14E but for somewhat increased extent with increasing developmental age.
Abbreviations: C: cortex; Cap: capsule; CT: connective tissue; Ep: epithelium; ETOC: embryonic thymus organ culture; KNC/KNA: keratin-negative cells/areas; M: medulla; MΦ: macrophages; PVS: perivascular space; Tb: trabeculae; Th: thymocytes.

negative at all time points tested. MUI-83 is an mAb reacting primarily with thymocytes (95% from 16E) (Fig. 3j) and a subpopulation of peripheral T cells in the adult chicken (Boyd et al., 1992; A. G. Bean, N. J. Davidson and R. L. Boyd, submitted). MUI-83$^+$ thymocytes were present at the onset of culture and expanded rapidly, peaking at approximately 85–90% by day 6 ETOC and were primarily located within the corticallike epithelial areas of the lobe, as in the embryo (Fig. 3h). In contrast to the embryonic thymus, the frequency of MUI-83$^+$ thymocytes had decreased markedly by days 10–12 ETOC (50% compared to 96% at 20E) and they were scattered throughout the epithelial and nonepithelial (keratin-negative) areas of the lobe. Section staining for the CD3, CD4, and CD8 antigens demonstrated a similar developmental trend for thymocytes in ETOC, correlating with that observed previously (Davidson et al., 1992).

### DISCUSSION

ETOC is a model whereby the functional significance of thymic stromal-cell antigens may be assessed by their presence (or absence), and, more definitively, by blocking molecular interactions through addition of purified mAb and assessing the subsequent effects on developing thymocytes. Such a model may also be used to analyze mechanisms of thymocyte selection with respect to antigens present during embryonic life. We have previously shown that thymocytes proliferate and differentiate in chicken ETOC in a manner analogous to that in the embryo (Davidson et al., 1992), implying that the stromal microenvironment is sufficiently intact and functional *in vitro*. As a prologue to identifying stromal antigens of functional importance, the maintenance and development of stromal subsets defined by mAb was delineated in ETOC and compared to normal embryonic development.

Both cortical and medullary epithelial cells were present in ETOC for up to 8 days, similar to that in the embryo, as identified morphologically by the anticytokeratin reagent and MUI-54, the latter a pan epithelial marker in the thymus and possibly a marker of endodermal epithelium (Wilson et al., 1992). Furthermore, epithelial cells in ETOC expressed antigens normally present in the cortex and/or medulla of the embryonic thymus, although their distribution/organization was not always identical. Similarly, in murine FTOC, cortical and medullary epithelial cells have been detected using specific mAb (van Vliet et al., 1985; D. I. Godfrey, G. A. Waanders, D. J. Izon, C. L. Tucek and R. L. Boyd, in preparation). Antigens present on type I epithelium, which normally lines the capsule, trabeculae, and perivascular spaces (the latter predominantly in the medulla), were present at a much higher frequency in ETOC compared to the embryo and lacked the organization seen in the latter. This disorganization, observed beyond day 4 ETOC, may be due to the lack of a functional vascular system, as demonstrated by an absence of medullary vascular endothelial cells defined by MUI-82. During normal thymic histogenesis, mesenchymal tissue accompanies vascular penetration of the epithelial rudiment, resulting in the formation of trabeculae and perivascular spaces (Le Douarin and Jotereau, 1975). It would appear that as these structures do not form in ETOC, the epithelium normally associated with them is not restricted to defined regions. The extensive expression of the normally infrequent stromal cell molecule detected by MUI-52 suggests either a high incidence of this cell type or that this molecule is upregulated *in vitro*. In support of the latter, high levels of expression are also noted in epithelial monolayer culture (R. L. Boyd and T. W. Wilson, unpublished observations). Additionally, this marker is expressed by bursal follicle-associated epithelium from 15E to hatching, a time when lymphoid precursors are recruited to, and localize in, the bursal follicles (Wilson and Boyd, 1991).

Medullary epithelial components, defined by MUI-62, were present throughout the ETOC period, although at a lower frequency compared to the embryo. A similar trend was noted for epithelial cells/clusters defined by MUI-66, MUI-72, and MUI-80, which normally include medullary epithelium in their distribution. This was particularly noticeable by days 10–12, when these molecules were largely restricted to keratin-negative cells. It may be, therefore, that medullary components are underdeveloped in ETOC and that the extensive reactivity seen with such markers as MUI-52, -51, -53, and -70 is due to an overrepresentation of subcapsular/subtrabecular and cortical epithelium. Similarly, the subcapsular/corticallike reactivity of MUI-58 is more frequent in ETOC. This mAb demonstrates the phenotypic relationship between subcapsular and medullary epithelium (Boyd et al., 1992), a relationship conserved between birds and mammals (Haynes, 1984; Colic et al., 1988; Godfrey et al., 1990; Izon and Boyd, 1990). As the correct development of the medulla appears to require the presence of mature, $TcR^+$ thymocytes (Shores et al., 1991), a low frequency of $CD4^+$ mature cells in cultured thymus (Davidson et al., 1992) may result in underdevelopment of the medulla. Alternatively, epithelial cells not normally expressing cortical antigens may do so in ETOC.

Lampert and Ritter (1988) have proposed that both cortical and medullary epithelium are derived from a resident epithelial stem cell. MUI-66 may be a marker for such stem cells as it is a pan epithelial marker at 10E, becoming restricted, with increasing developmental age, to isolated cortical epithelium and a subset of medullary epithelial clusters of a morphologically less differentiated nature, being round-bodied. It also stains the least differentiated epithelial cell layers in the skin (Boyd et al., 1992). The maintenance of its pan epithelial reactivity in ETOC suggests that epithelial maturation may be slowed. This is also consistent with the concept that cortical epithelium is of a simple or less differentiated form than medullary epithelium on the basis of expression of different isoforms of cytokeratin (Colic et al., 1989; reviewed Brekelmans and van Ewijk, 1990) and mAb reactive with cortical epithelium showed increased reactivity/distribution in ETOC.

Additionally, MUI-66, -72, and -80, as well as MUI-56 (probably a marker of mesodermally derived tissue (Wilson et al., 1992), demonstrated the increasing proportion of keratin-negative cells in ETOC, which comprised at least half of the lobe by day 12, consisting of fibroblasts, and, to a lesser extent, MΦ and dendritic cells (Boyd et al., 1992). Although MΦ are present in the 10E thymus (Oliver and Le Douarin, 1984), neither MUI-79 nor MUI-36, both reactive with a subset of MΦ (as yet there is no chicken pan-macrophage reagent available), showed a significantly increased frequency of MΦ reactivity over that seen in the embryo. The proportion of keratin-negative tissue in ETOC expanded with time in culture, often present as large, circular areas both within and around the epithelial regions, predominating by days 10–12 ETOC. Similarly, although to a lesser extent, keratin-negative cells, including reticular fibroblasts, were observed within and around epithelial areas in murine FTOC (van Vliet et al., 1985).

There was extensive expression of MHC class II molecules, as defined by MUI-78, in ETOC. Up to day 6 ETOC, MUI-78 showed cortical and medullary reactivity comparable to that in the embryo. In the later stages of culture, however, confluent reactivity was often seen over the entire cultured lobe area consisting of at least 50% nonepithelial cells. It is difficult to make a comparison with MHC class II expression in murine FTOC as reports are conflicting, some authors claiming it is low (Lo and Sprent, 1986), and others have observed strong development *in vitro* (Jenkinson et al., 1981). A granular staining pattern was often observed with MUI-78, suggestive of secreted MHC II. This may account for a twofold higher proportion of MUI-78$^+$ thymocytes in ETOC (data not shown) that may acquire MHC molecules from their surrounds (Sharrow et al., 1981). Whether the expression of MHC class II by most nonepithelial cells toward the end of the culture period is also due to passive acquisition is not known. It is possible that these nonepithelial cells, which are predominantly MUI-56$^+$ fibroblasts, may express class II determinants in response to locally secreted cytokines such as $\gamma$-interferon, previously demonstrated to regulate the class II expression of cultured epithelial cells (Berrih et al., 1985), although there is as yet no direct evidence for the presence of $\gamma$-interferon in ETOC.

An antigen shared between stromal cells, B cells, and thymocytes is defined by MUI-36. Expression of this marker was upregulated on thymocytes during ETOC (half of which coexpressed TcR/CD3 complex; data not shown). Some reactivity could be attributed to MΦ, but not to B cells, which do not appear to develop in ETOC. This suggests that either B-cell precursors were not present in the first wave of precursor cells entering the thymus at 6.5E (Coltey et al., 1987) and/or the lack of suitability of the embryonic thymus in promoting B-cell development and/or maintenance *in vitro*. Such data are also consistent with the paucity of B cells in the normal embryonic thymus (N. J. Davidson and A. G. Bean, unpublished data).

In summary, cortical and medullary epithelial and nonepithelial cell antigens, including MHC class II, developed and were maintained in ETOC, reflecting that in the embryo within a limited time frame. However, the data suggest that epithelial maturation may be slowed *in vitro*, whereas conditions appear permissive for the maintenance of nonepithelial cells, perhaps as their requirements are less stringent than those of epithelial cells. Concurrent with the decreasing proportion of epithelial areas and an increasing proportion of keratin-negative tissue was a decline in thymocyte frequency, demonstrated by MUI-83 reactivity, and as previously documented using mAb reactive with standard T-cell determinants (Davidson et al., 1992). It has been similarly demonstrated during culture of rat thymic fragments that a type of dedifferentiation of epithelial cells is coincident with a depletion of thymocytes (Kendall et al., 1988). In this model, after days 6–9 of culture, all epithelial cells within the thymic fragment possess a uniform and unusual morphology that, following *in vivo* reconstitution of the fragment with host thymocytes, "redifferentiates" into distinct cortical and medullary compartments. It would be particularly interesting to determine if such a pattern of cellular reorganization would occur following grafting of the cultured chicken thymus into embryonic recipients. This data, combined with that presented herein, would suggest that an intimate relationship exists between developing T cells and the maintenance and/or maturation of epithelial cells, a conclusion that is supported by the recent finding that normal T cells are able to restore the thymic stromal microenvironment of scid mice (Shores et al., 1991; Surth et al., 1992). Beyond day 8 ETOC, the constraints of the system increasingly effect cellular development, particularly that of the epithelium. Such constraints include a decreased efficiency of nutrient/gaseous exchange with increasing lobe size (Jenkinson and Owen, 1990) and a lack of second-wave thymocyte and stromal precursors (MΦ and dendritic cells) at 12E (Oliver and Le Douarin, 1984; Coltey et al., 1987), the interaction of which with the developing stroma appears necessary for continued growth, as has been demonstrated in scid mice (Shores et al., 1991).

Hence, with respect to both stromal (this study) and thymocyte development (Davidson et al., 1992), thymopoiesis in chicken ETOC reflects that in the embryo within a limited time frame (0–8 days) and represents a useful model with which to examine the functional potential of thymic microenvironmental components. Furthermore, the thymic development described in this study is reminiscent of that in murine FTOC (van Vliet et al., 1985; D. I. Godfrey, G. A. Waanders, D. J. Izon, C. L. Tucek and R. L. Boyd, in preparation) and consistent with the high degree of similarity of thymic events between the two species (reviewed in Cooper et al., 1991). This model is currently proving useful in identifying functionally relevant thymic stromal molecules and to address the issue of the symbiotic relationship between developing thymocytes and stromal-cell subsets.

### MATERIALS AND METHODS

### Chickens

Closed colony Australorp/White Leghorn F1 hybrid chicks and embryos were obtained from Research Poultry Farm (Research, Australia). Chicks were kept under standard animal house conditions and eggs maintained in a humidified incubator at 39°C, age estimated by the duration of incubation.

### Embryonic Thymus Organ Culture

The method for ETOC has been previously detailed (Davidson et al., 1992). Briefly, thymus lobes from 10-day-old embryos were isolated under sterile conditions and 5–8 lobes cultured on 0.45 $\mu$m polysulfone filter membranes (Gelman Sciences, Michigan) supported by gelatin sponge (Upjohn, Kalamazoo, Michigan) in RPMI-1640 tissue-culture medium (supplemented with 10% [v/v] heat-inactivated fetal calf serum and 2 mM L-glutamine) for 2–12 days in a humidified, 42°C incubator. Medium was replaced after 6 days of culture.

### Antibodies and Conjugates

Thymic stromal cell reactive mAb were produced in our laboratory and have been described in detail elsewhere (Boyd et al., 1991; Wilson et al., 1992). These were used as undiluted hybridoma supernatants and revealed with a fluorescein isothiocyanate (FITC)-conjugated $F(ab')_2$ fragment of affinity-purified sheep anti-mouse immunoglobulin (Silenus Laboratories, Melborne). Rabbit anti-cytokeratin immunoglobulin (wide spectrum; Dakopatts, Santa Barbara, California), used to identify epithelial cells, was revealed with tetramethyl rhodamine isothiocyanate (TRITC) conjugated sheep anti-rabbit immunoglobulin (Silenus).

### Tissue Section Staining

Tissue sections of 10E thymus lobes cultured for 0, 2, 4, 6, 8, 10, and 12 days were examined and compared to age-matched control thymus; 10, 12, 14, 16, 18, 20E, and 1 day posthatching. Organ-cultured thymus lobes were covered in Tissue-Tek embedding compound (Miles Scientific, Elkhart, Indiana) and snap frozen in plastic "boats" on the surface of liquid nitrogen while still on the filter membranes upon which they were cultured, as their removal often resulted in damage to the lobe structure. Thymus lobes from embryos or chicks were removed, trimmed of excess fat and connective tissue, immersed in Tissue-Tek, and snap frozen as before. Cryostat cut sections (4 $\mu$m) of cultured (cut parallel to the plane of the filter membrane) and control thymus were air dried on gelatinized glass slides and stained using standard double-labeling immunofluorescence techniques. Briefly, sections were incubated simultaneously with both mAb supernatant and anti cytokeratin, washed by immersion in phosphate buffered saline (3×5 min, with shaking), and then incubated with a mixture of FITC-conjugated sheep anti-mouse immunoglobulin and TRITC-conjugated anti-rabbit immunoglobulin. Sections were then washed and mounted under glass coverslips with veronal buffered glycerol (pH 8.6) and examined using a Zeiss Axioskop epi-fluorescence microscope. Photomicrographs were recorded using a Zeiss MC100 camera using AGFA XRS 100/speed professional film, with exposure time determined by the degree of intensity of fluorescence staining.

### ACKNOWLEDGMENTS

This work was supported by grants from the Australian National Health and Medical Research Council (860396), the Anti-Cancer Council of Victoria (131658), and the Monash University Special Research Grant Scheme. The authors would like to thank Dr. Dale Godfrey for helpful discussion and his efforts and those of Elise Randle in reviewing the manuscript.

*(Received July 12, 1991)*

*(Accepted November 12, 1992)*

### REFERENCES

Berrih S., Arenzana-Seisdedos F., Cohen S., Devos R.,

Charron D., and Virelizier J.L. (1985). Interferon-γ modulates HLA class II antigen expression on cultured human thymic epithelial cells. J. Immunol. **135:** 1165–1171.

Boyd R.L., Bean A.G.D., Wilson T.J., Ward H.A., and Gershwin M.E. (1992). Phenotypic characterization of chicken thymic stromal elements. Dev. Immunol. **2:** 51–66.

Boyd R.L., and Hugo P. (1991). Towards an integrated view of thymopoiesis. Immunol. Today **12:** 71–79.

Boyd R.L., Wilson T.J., van de Water J., Haapanen L.A., and Gershwin M.E. (1991). Selective abnormalities in the thymic microenvironment associated with avian scleroderma, an inherited fibrotic disease of L200 chickens. J. Autoimmunity **4:** 369–380.

Brekelmans P., and van Ewijk W. (1990). Phenotypic characterization of murine thymic microenvironments. Semin. Immunol. **2:** 13–24.

Carding R., Hayday A.C., and Bottomly K. (1991). Cytokines in T cell development. Immunol. Today **12:** 239–245.

Colic M., Jovanovic S., Mitrovic S., and Dujic A. (1989). Immunohistochemical identification of six cytokeratin-defined subsets of the rat thymic epithelial cells. Thymus **13:** 175–185.

Colic M., Matanovic D., Hegedis L., and Dujic A. (1988). Immunohistochemical characterization of rat thymic nonlymphoid cells. I. Epithelial and mesenchymal components defined by monoclonal antibodies. Immunology **65:** 277–284.

Coltey M., Jotereau F. V., and Le Douarin N.M. (1987). Evidence for a cyclic renewal of lymphocyte precursor cells in the embryonic chick thymus. Cell Differ. **22:** 71–82.

Cooper M.D., Chen C.H., Bucy R.P., and Thompson C.B. (1991). Avian T cell ontogeny. Adv. Immunol. **50:** 87–117.

Cosgrove D., Gray D., Dierich A., Kaufman J., Lemeur M., Benoist C., and Mathis D. (1991). Mice lacking MHC class II molecules. Cell **66:** 1051–1066.

Davidson N.J., Chen C.L., and Boyd R.L. (1992). Kinetics of chicken embryonic thymocyte development in ovo and in organ culture. Eur. J. Immunol. **22:** 1429–1435.

Godfrey D.I., Izon D.J., Tucek C.L., Wilson T.J., and Boyd R.L. (1990). The phenotypic heterogeneity of mouse thymic stromal cells. Immunology **70:** 66–74.

Guillemot F.P., Oliver P.D., Peault B.M., and Le Douarin N.M. (1984). Cells expressing Ia antigens in the avian thymus. J. Exp. Med. **160:** 1803–1891.

Haynes B.F. (1984). The human thymic environment. Adv. Immunol. **36:** 87–142.

Izon D.J., and Boyd R.L. (1990). The cytoarchitecture of the human thymus detected by monoclonal antibodies. Human Immunol. **27:** 16–32.

Jenkinson E.J., and Owen J.J.T. (1990). T-cell differentiation in thymus organ cultures. Semin. Immunol. **2:** 51–58.

Jenkinson E.J., van Ewijk W., and Owen J.J.T. (1981). Major histocompatibility complex antigen expression on the epithelium of the developing thymus in normal and nude mice. J. Exp. Med. **153:** 280–292.

Kampinga J., Berges S., Boyd R.L., Brekelmans P., Colic M., van Ewijk W., Kendall M.D., Ladyman H., Nieuwenhuis P., Ritter M.A., Schurrman H.J., and Tournefier A. (1989). Thymic epithelial antibodies: Immunohistological analysis and introduction of nomenclature. Summary of the epithelium workshop held at the 2nd workshop "The thymus. Histophysiology and dynamics in the immune system." Thymus **13:** 165–173.

Kendall M.D., Schurrman H.J., Fenton J., Broekhuizen R., and Kampinga J. (1988). Implantation of cultured thymic fragments in congenitally athymic (nude) rats. Cell Tissue Res. **254:** 283–294.

Lampert I.A., and Ritter M.A. (1988). The origin of the diverse epithelial cells of the thymus: Is there a common stem cell? In: Thymus Update 1. The microenvironment of the human thymus, Kendall M.D. and Ritter M.A. Eds. (London: Harwood Academic Publishers), pp. 5–25.

Le Douarin N.M., and Jotereau F.V. (1975). Tracing of cells of the avian thymus through embryonic life in interspecific chimeras. J. Exp. Med. **142:** 17–40.

Lo D., and Sprent J. (1986). Exogenous control of I-A expression in fetal thymus explants. J. Immunol. **137:** 1772–1775.

Oliver P.D., and Le Douarin N.M. (1984). Avian thymic accessory cells. J. Immunol. **132:** 1748–1755.

Sharrow S.O., Mathieson B.J., and Singer A. (1981). Cell surface appearance of unexpected host MHC determinants on thymocytes from radiation bone marrow chimeras. J. Immunol. **126:** 1327–1335.

Shores E.W., van Ewijk W., and Singer A. (1991). Disorganization and restoration of thymic medullary epithelial cells in T-cell receptor negative scid mice: Evidence that receptor-bearing lymphocytes influence maturation of the thymic microenvironment. Eur. J. Immunol. **21:** 1657–1661.

Surth C.D., Ernst B., and Sprent J. (1992). Growth of epithelial cells in the thymic medulla is under the control of mature T cells. J. Exp. Med. **176:** 611–616.

Takeuchi T., Kubonishi I., Ohtsuki Y., and Miyoshi I. (1991). A new monoclonal antibody to human subcapsular thymic epithelial cells. Virchows Archiv. A Pathol. Anat. **419:** 147–151.

Van de Wijngaert F.P., Kendall M.D., Schurrman H.J., Rademakers L.H.P.M., and Kater L. (1984). Heterogeneity of epithelial cells in the human thymus. An ultrastructural study. Cell Tissue Res. **237:** 227–237.

Van Vliet E., Jenkinson E.J., Kingston R., Owen J.J.T., and van Ewijk W. (1985). Stromal cell types in the developing thymus of the normal and nude mouse embryo. Eur. J. Immunol. **15:** 675–681.

Wilson T.J., and Boyd R.L. (1991). The ontogeny of chicken bursal stromal cells defined by monoclonal antibodies. Dev. Immunol. **1:** 31–39.

Wilson T.J., Davidson N.J., Boyd R.L., and Gershwin M.E. (1992). Phenotypic analysis of the chicken thymic microenvironment during ontogenic development. Dev. Immunol. **2:** 19–27.

Zijlstra M., Bix M., Simister N.E., Loring J.M., Raulet D.H., and Jaenisch R. (1990). β2-microglobulin deficient mice lack $CD4^{-}8^{+}$ cytolytic T cells. Nature **344:** 742–746.

